# 4-Methylumbelliferone enhances the effects of chemotherapy on both temozolomide-sensitive and resistant glioblastoma cells

**DOI:** 10.1038/s41598-023-35045-3

**Published:** 2023-06-08

**Authors:** Matías A. Pibuel, Daniela Poodts, Sofía A. Sias, Agustín Byrne, Silvia E. Hajos, Paula G. Franco, Silvina L. Lompardía

**Affiliations:** 1 Cátedra de Inmunología, Departamento de Microbiología, Inmunología y Biotecnología, Facultad de Farmacia y Bioquímica, Instituto de Estudios de la Inmunidad Humoral (IDEHU)- CONICET, Universidad de Buenos Aires, Junín 956 4° Piso, 1113 Capital Federal, Argentina; 2 Departamento de Química Biológica, Facultad de Farmacia y Bioquímica, Instituto de Química y Fisicoquímica Biológicas (IQUIFIB)-CONICET, Universidad de Buenos Aires, 1113 Capital Federal, Argentina

**Keywords:** Cancer therapy, CNS cancer

## Abstract

Glioblastoma (GBM) is the most frequent malignant primary tumor of the CNS in adults, with a median survival of 14.6 months after diagnosis. The effectiveness of GBM therapies remains poor, highlighting the need for new therapeutic alternatives. In this work, we evaluated the effect of 4-methylumbelliferone (4MU), a coumarin derivative without adverse effects reported, in combination with temozolomide (TMZ) or vincristine (VCR) on U251, LN229, U251-TMZ resistant (U251-R) and LN229-TMZ resistant (LN229-R) human GBM cells. We determined cell proliferation by BrdU incorporation, migration through wound healing assay, metabolic and MMP activity by XTT and zymography assays, respectively, and cell death by PI staining and flow cytometry. 4MU sensitizes GBM cell lines to the effect of TMZ and VCR and inhibits metabolic activity and cell proliferation on U251-R cells. Interestingly, the lowest doses of TMZ enhance U251-R and LN229-R cell proliferation, while 4MU reverts this and even sensitizes both cell lines to TMZ and VCR effects. We showed a marked antitumor effect of 4MU on GBM cells alone and in combination with chemotherapy and proved, for the first time, the effect of 4MU on TMZ-resistant models, demonstrating that 4MU would be a potential therapeutic alternative for improving GBM therapy even on TMZ-refractory patients.

## Introduction

Glioblastoma (GBM) is the most frequent malignant primary tumor of the central nervous system (CNS), constituting 50% of all diagnosed gliomas with an incidence that increases with age. It is classified as a CNS tumor WHO grade 4^1^ and causes high mortality, with a median survival of only 14.6 months after diagnosis, even under therapy. Moreover, GBM patients present a 5-year survival rate of 20% for individuals younger than 14 and 6% for those older than 75 years^2^. The aggressiveness of GBM lies in its exacerbated migration rate and the subsequent invasion of the surrounding brain tissue.

The current protocol of care is based on surgical resection, radiotherapy and adjuvant chemotherapy, being the alkylating agent temozolomide (TMZ) the first-line drug^3^. Together with TMZ, three other drugs are approved by the FDA for GBM therapy: Lomustine, Bevacizumab, and Carmustine^3,4^. However, after TMZ implementation in 2005, there has been no improvement in GBM treatment, except for the addition of Bevacizumab, which increased only 2 months of the progression-free survival time, but not the overall survival^3,5^. Therefore, it is imperative to find new therapeutic alternatives, since 50% of total GBM patients are refractory to TMZ treatment, which exhibits several adverse effects, such as myelosuppression and hepatotoxicity^6–9^. This resistance is mainly due to the overexpression of the enzyme Methyl Guanine Methyl Transferase (MGMT) and by acquired TMZ resistance mechanisms^6–10^. Under this scenario, there are only a few drug cocktails under evaluation that combine Lomustine, Procarbazine and Vincristine (VCR), or Irinotecan, TMZ plus VCR, among others^3,4^. However, these therapies still lack consensus, and their benefits are yet being questioned^11–13^. In this way, the combination of currently used drugs with new alternatives poses an interesting approach^14^. On the other hand, drug repositioning and the use of natural compounds with large effects have been explored for some years and are currently gaining significance^15–21^. Specifically, 4-methylumbelliferone (4MU) is a natural coumarin derivative without adverse effects reported, that is commercially available as “hymecromone”, as it is used in Europe and Asia as a choleretic and antispasmodic drug for treating biliary spasm. Its effects on other pathologies, such as autoimmune diseases, cancer and even COVID-19 are being explored^22–24^. 4MU is a known hyaluronan (HA) synthesis inhibitor, via the inhibition of HA synthases expression and depletion of their substrate, UDP-glucuronic acid. Recently, it was demonstrated that oral administration of 4MU in healthy patients diminished HA content both in serum and sputum, showing that its effect on HA metabolism is systemically relevant^25–27^. Lately, our and other groups have shown that 4MU exerts some of its effects in an HA-independent manner, highlighting its potential as an alternative therapy. In this way, 4MU diminishes cell proliferation, migration and induces antitumor mechanisms, such as apoptosis and senescence, in a wide variety of cancer types, including ovarian, breast, hepatocellular, pancreatic, prostate, fibrosarcoma, chronic and acute myeloid leukemia, osteosarcoma, malignant pleural mesothelioma, among others^22,28–39^. Previously in our lab, we determined the antitumor effects of 4MU on a murine and two human GBM cell lines, which mainly consisted of inhibition of cell proliferation, induction of apoptosis in the murine model, and induction of senescence and inhibition of migration in the human model^40,41^. Furthermore, a recent publication showed the efficacy of 4MU in an in vivo GBM model^42^.

In the present work, we examined the effect of 4MU and TMZ combination on the proliferation, migration, metabolic and MMP activity as well as cell death induction on U251 and LN229 human GBM cells. Furthermore, we studied the effect of the combination of 4MU with VCR on metabolic activity and cell proliferation in both GBM cell lines. Finally, we developed the U251-TMZ (U251-R) and LN229-TMZ (LN229-R) resistant cell lines and assessed the efficacy of 4MU alone and in combination with TMZ, demonstrating that 4MU markedly sensitized these models to TMZ effects.

Surprisingly, our results suggest that the use of TMZ in patients with resistance to treatment not only lacks antitumor effects, but it could be detrimental since this drug increases the proliferation of the resistant GBM cell lines. This finding could represent a counterproductive situation for TMZ-non-responding patients, and it should be deeply studied. Overall, the use of 4MU would be a potential therapeutic alternative for improving GBM therapy even in TMZ-refractory patients.

## Results

### 4MU exerts similar effects as TMZ on GBM cells regarding metabolic activity, proliferation and cell death

**Figure 1:**
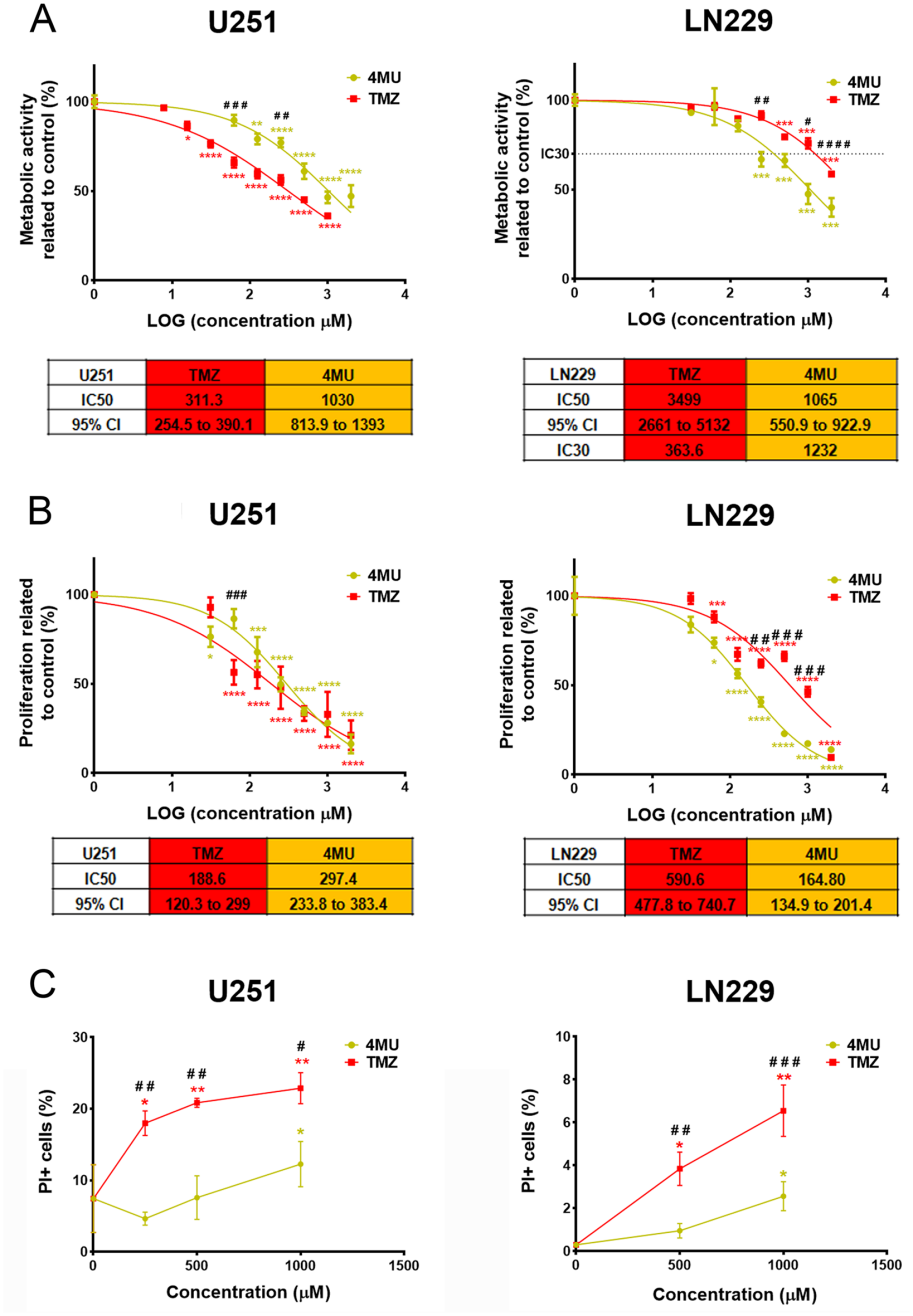
Effect of TMZ and 4MU on metabolic activity, proliferation and cell death. **(A)** Metabolic activity was determined by XTT assay after 72 h of treatment with either TMZ or 4MU. Results are expressed as the percentage of Abs (n = 3) in relation to vehicle control, as described in the “Materials and methods” section. The dose–response curve is shown for each cell line, along with IC50 values and its 95% CI for each treatment. **(B)** Cell proliferation was determined by BrdU incorporation and ELISA-like assay after 72 h of treatment with either TMZ or 4MU. Results are expressed as the percentage of Abs (n = 3) in relation to vehicle control, as described in the “Materials and methods” section. The dose–response curve is shown for each cell line, along with IC50 values and its 95% CI for each treatment. **(C)** Cell death was determined by FDA/PI staining and flow cytometry after 72 h of treatment with either TMZ or 4MU. Results are expressed as the percentage of PI + cells (n = 3) in relation to the vehicle control. In all graphs, each dot represents the mean ± SD of at least 3 independent experiments. Asterisks (*) over each dot indicate differences between treated cells and vehicle control cells. Hash sign (#) over each dot indicates differences between TMZ treated cells vs cells treated with the same dose of 4MU. */^#^P < 0.05, **/^##^P < 0.01, ***/^###^P < 0.001, ****/^####^P < 0.0001 and ns = nonsignificant (P > 0.05).

Considering that TMZ is the first-line drug in glioblastoma therapy and that in our previous work we demonstrated the effect of 4MU on glioblastoma cells^41^, we aimed to compare the effect of such drug with TMZ. For this purpose, U251 and LN229 cells were treated with either TMZ or 4MU and XTT, BrdU incorporation and cell death by flow cytometry (FC) staining with PI assays were performed. As shown in Fig. 1A, 4MU exhibits a greater inhibition of LN229 cell’s metabolic activity than TMZ after 72 h of treatment, opposite to what is seen on U251 cells. Considering that the IC50 for 4MU in both cell lines is similar, the differences observed would be attributed to each cell line response to TMZ. Similar results were obtained on metabolic activity after 48 h of treatment (see Supplementary Fig. S1). It is worth noting that, in the case of LN229 cells, the IC50 was mathematically inferred by extrapolation, given that no treatment reached 50% of inhibition. Therefore, a real experimental value reached for all conditions (in this case 30% inhibition) was considered and the IC30 was calculated.

Figure 1B shows that the inhibitory effect of 4MU on cell proliferation was higher than that of TMZ on the LN229 cell line, opposite to what is seen on U251 cells. Again, the effect of 4MU on both cell lines was similar while a higher variation was observed on the TMZ effect. After obtaining the IC50 for each curve, we observed a 3.6-fold decrement in such parameter, demonstrating the greater potency of 4MU compared with the first-line drug on LN229 cells.

Finally, we performed the PI staining to evaluate cell death and showed that only the dose of 1000 μM TMZ induces cell death in more than 20% of U251 cells (Fig. 1C).

Overall, these results demonstrate that 4MU has a significant and consistent inhibitory effect along both GBM cells, while the effects of TMZ seem to be dependent on the cell line.

### 4MU sensitizes GBM but not NBPC cells to TMZ effects

**Figure 2:**
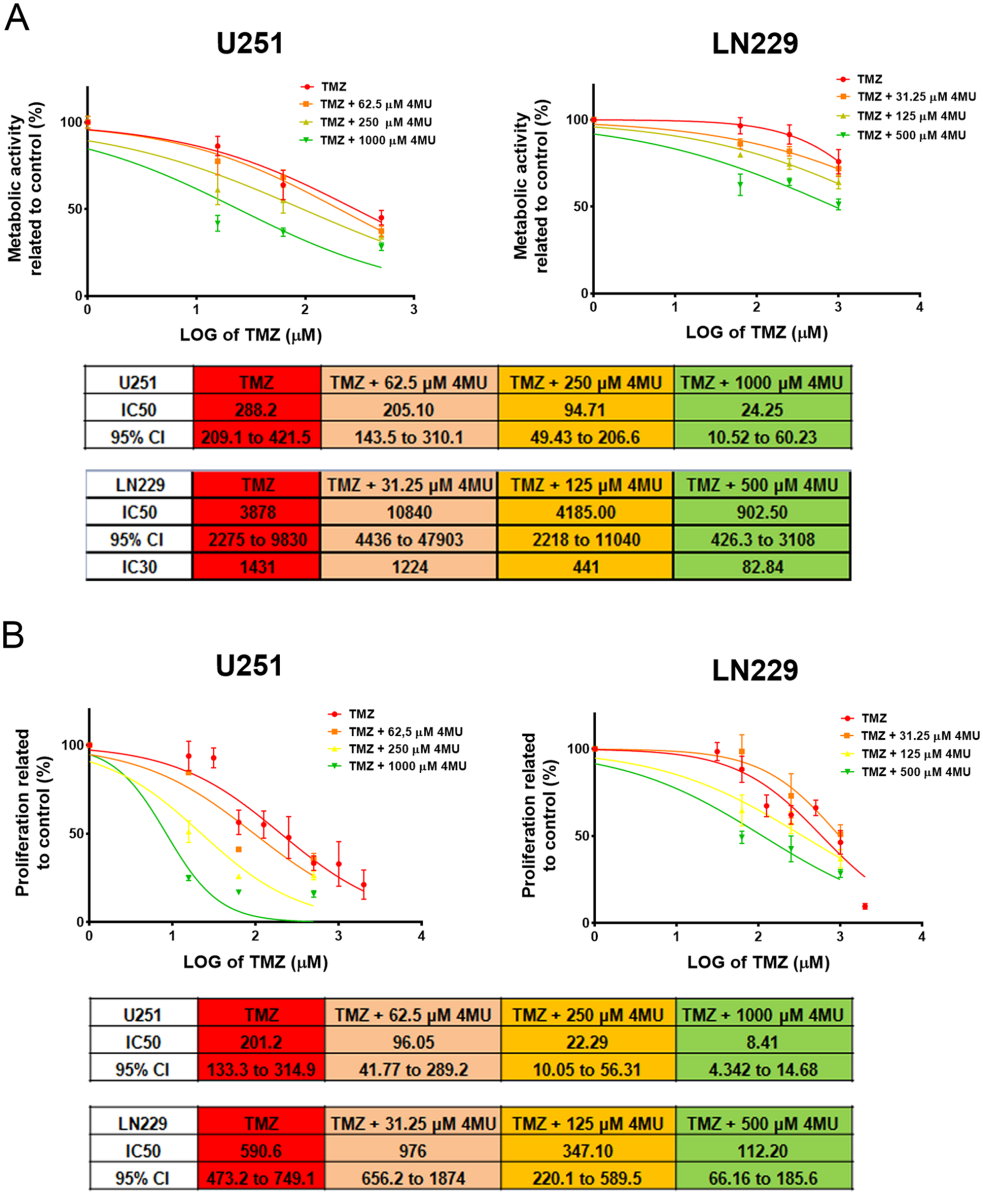

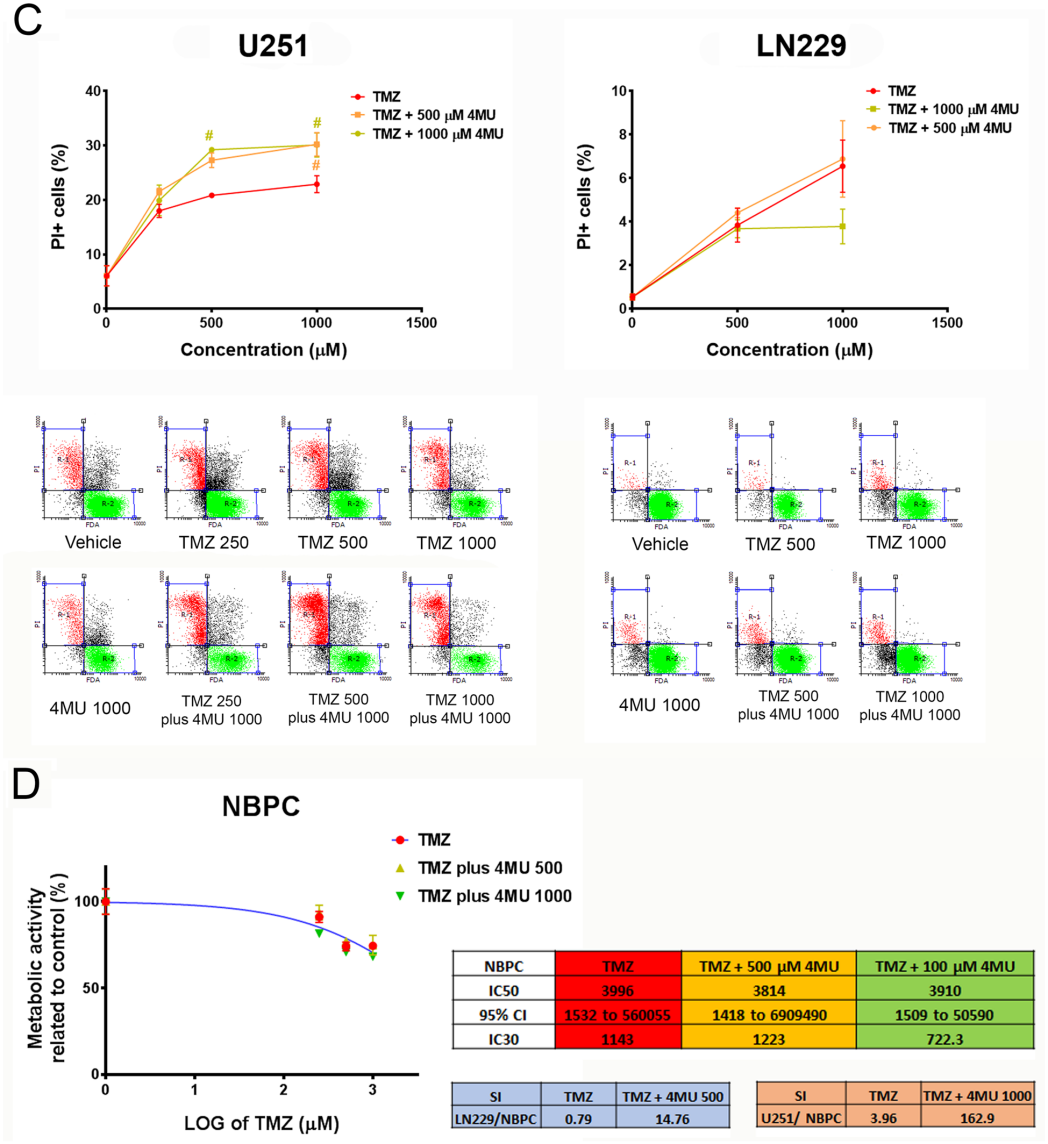
Effect of TMZ and its combination with 4MU on metabolic activity, proliferation and cell death. **(A)** Metabolic activity was determined by XTT assay after 72 h of treatment with TMZ alone or combined with 4MU. Results are expressed as the percentage of Abs (n = 3) in relation to vehicle control, as described in the “Materials and methods” section. The dose–response curves are shown, along with IC50 values for each combination and its 95% CI. **(B)** Cell proliferation was determined by BrdU incorporation and ELISA-like assay after 72 h of treatment with TMZ alone or combined with 4MU. Results are expressed as the percentage of Abs (n = 3) in relation to vehicle control, as described in the “Materials and methods” section. The dose–response curves are shown, along with IC50 values for each combination and its 95% CI. **(C)** Cell death was determined by FDA/PI staining and flow cytometry after 72 h of treatment with TMZ alone or in combination with 4MU. Results are expressed as the percentage of PI + cells (n = 3) in relation to the vehicle control. Representative dot plots are shown under graphics. Hash sign (#) over each dot indicates differences between treated cells with TMZ + 4MU vs cells treated with the same dose of TMZ alone. **(D)** Metabolic activity was determined on a mouse NBPC through XTT assay after 72 h of treatment with TMZ alone or combined with 4MU. Results are expressed as the percentage of Abs (n = 3) in relation to vehicle control, as described in the “Materials and methods” section. The dose–response curves are shown, along with IC50 values for each combination and its 95% CI. The SI was calculated as described in “Materials and methods” section. ^#^P < 0.05, ^##^P < 0.01, ^###^P < 0.001, ^####^P < 0.0001 and ns = nonsignificant (P > 0.05). In all graphs, each dot represents the mean ± SD of at least 3 independent experiments.

Considering that 4MU and TMZ have different mechanisms of action, we decided to investigate the effect of the co-treatment on GBM cells. To perform these studies, different doses of TMZ and 4MU were chosen for their combination, specifically those that inhibited metabolic activity by 50%, 25%, and 5% for each cell line. First, we evaluated the effect on metabolic activity after 72 h of treatment using the XTT assay. As shown in Fig. 2A, the addition of 4MU to TMZ treatment enhances its effect on the metabolic activity of both cell lines. Using the calculated IC50, we observed that for the highest 4MU dose tested, this parameter decreased by 11.9 times on U251 cells and 4.3 times on LN229 cells. It is worth noting that in the case of LN229 cells, the IC50 was mathematically obtained by extrapolation, given that no treatment reached 50% of inhibition. Therefore, considering a real experimental value reached for all conditions (in this case, 30% of inhibition) we calculated the IC30, which showed a decrement of 17.3 times for LN229 cells. Similar analyses were performed for subsequent comparisons. Determination of metabolic activity after the treatment of both cell lines for 48 h revealed comparable results (see Supplementary Fig. S2). Then, we evaluated the effect of the co-treatment on cell proliferation by BrdU incorporation. Figure 2B shows that 4MU potentiated the effect of TMZ on cell proliferation in both cell lines. Moreover, the dose of TMZ required to reach 50% of inhibition decreased 38.3 times for U251 cells and 4.3 for LN229 cells. Furthermore, as is shown in Fig. 2C, 4MU enhanced the effect of TMZ on cell death on U251 but not on LN229 cells.

Finally, we performed an XTT assay to evaluate the effect of TMZ and 4MU combination on a mouse NBPC. Previously, we showed that 4MU does not modify the metabolic activity of a mouse NBPC^40^. As shown in Fig. 2D, 4MU did not sensitize the NBPC to the TMZ effect. In this regard, we calculated the in vitro selectivity index (SI). Interestingly, we observed values over 10 for the combination (4MU + TMZ) for both GBM cell lines (162.9 and 14.76 for U251 and LN229, respectively). Surprisingly, the SI was under 5 for TMZ alone (3.96 and 0.79 for U251 and LN229, respectively). These data suggest that the use of 4MU in combination with TMZ not only improves the efficacy of TMZ, but also the selectivity of the treatment.

All in all, these results highlight the potential of 4MU for improving the therapy in glioblastoma patients used as a co-adjuvant in TMZ treatment.

### 4MU enhances the TMZ effect on cell migration and MMP-2 activity on both U251 and LN229 cell lines

**Figure 3:**
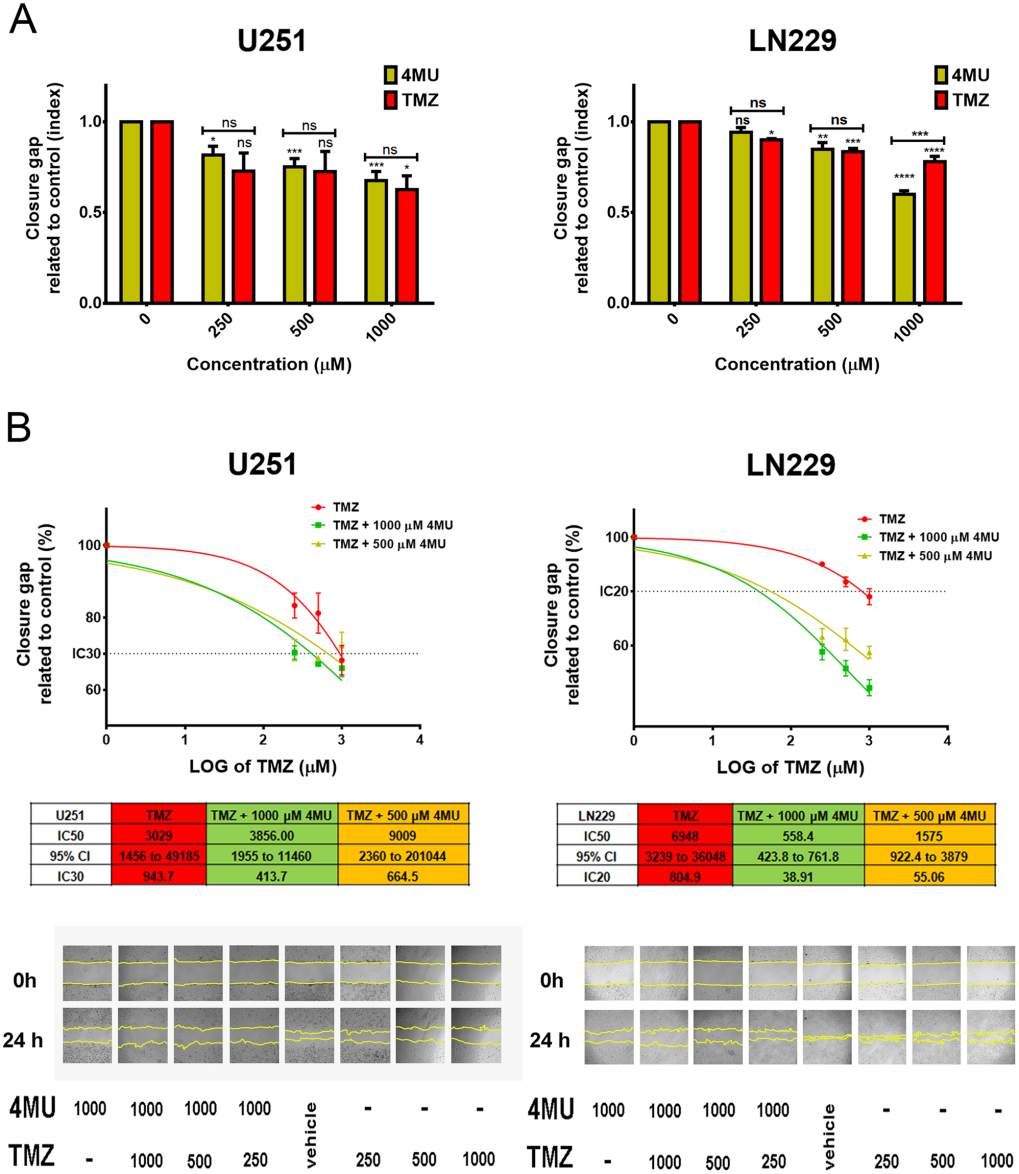

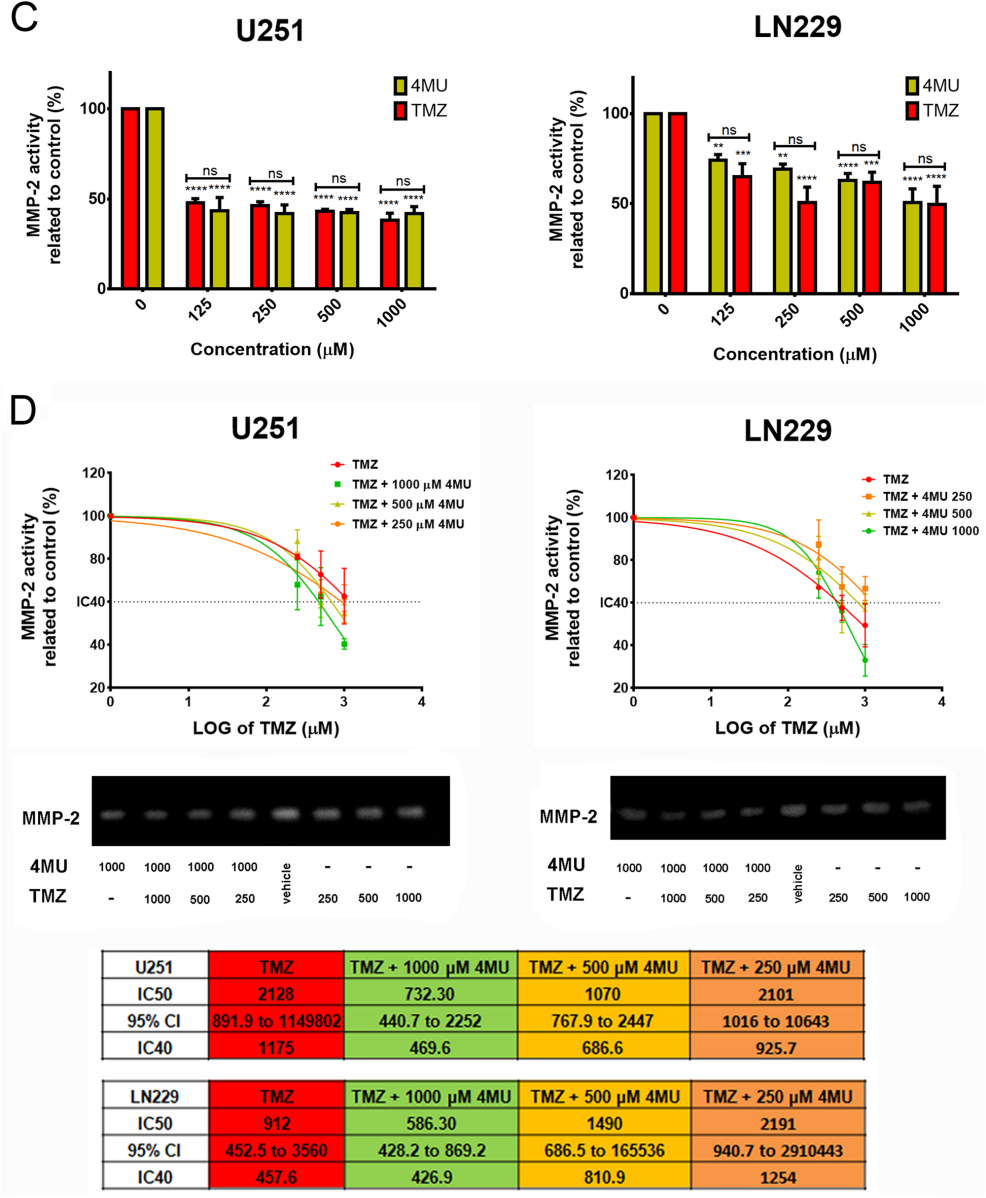
Effect of 4MU, TMZ and their combination on migration and MMP-2 activity. **(A,B)** U251 and LN229 cell lines were treated with either 4MU or TMZ **(A)** or their combination **(B)** and cell migration was determined by the wound healing assay after 24 h of treatment. The same wound area was photographed at 0 and 24 h. Results are expressed as closure gap index (n = 3), calculated as described in “Materials and methods” section. Results are expressed as closure gap index (n = 3) in relation to vehicle control, as described in the “Materials and methods” section. Representative photographs are shown below the tables. Yellow lines show the edges of migrating cells at 0 and 24 h. The dose–response curves for the combined treatments are shown, along with IC50 values for each combination and its 95% CI. **(C,D)** MMPs activity of U251 and LN229 cells after 24 h of treatment with either 4MU or TMZ **(C)** or their combination **(D)** was determined by zymography. The gelatinolytic activity was calculated as the percentage of densitometry values (n = 4) of bands in relation to vehicle control cells. A representative photograph is shown below the graph. The dose–response curves for the combined treatments are shown, along with IC50 values for each combination and its 95% CI. In all graphs, each dot or bar represents the mean ± SD of at least 3 independent experiments. Asterisks (*) over each bar indicate differences between treated cells and vehicle control cells. Asterisks over lines indicate differences between the indicated treatments. *P < 0.05, **P < 0.01, ***P < 0.001, ****P < 0.0001 and ns = nonsignificant (P > 0.05).

Considering that cell migration and MMPs activity are two features closely related to GBM malignancy, we performed the wound healing assay and gelatin zymography to evaluate the effect of the combination of 4MU and TMZ on such processes. First, we performed the wound healing assay to assess the effect of a 24 h treatment with each drug. As shown in Fig. 3A, 4MU and TMZ similarly decreased cell migration on both U251 and LN229 cell lines. Notably, the highest dose of 4MU diminished cell migration more efficiently than TMZ on LN229 cells.

Interestingly, 4MU enhanced the inhibition of cell migration exerted by TMZ on both cell lines (Fig. 3B). Furthermore, considering the IC30 for U251 and the IC20 for LN229, the highest doses of 4MU decreased such parameters by 1.4- and 14.6-folds, respectively. We performed a BrdU incorporation assay in the same conditions as the wound healing assay and corroborated that the changes in migration were not due to changes in proliferation (see Supplementary Fig. S3).

Then, we evaluated the effect of each drug on MMPs activity by performing zymography assays. We observed that 4MU and TMZ similarly diminished the MMP-2 activity (Fig. 3C). In accordance with the results obtained for cell migration, combining 4MU plus TMZ inhibits MMP-2 activity more efficiently than TMZ alone (Fig. 3D). Moreover, the combination of TMZ with 1000 μM of 4MU diminished the IC50 value for both cell lines, decreasing this parameter by 1.5 times for LN229 cells compared to TMZ alone and allowing it to reach this value in U251 cells.

These results reinforce the use of 4MU as a TMZ-adjuvant drug on GBM cells, not only to enhance cytotoxicity but also to inhibit the processes responsible for GBM aggressiveness.

### 4MU enhances the effect of VCR on metabolic activity and cell proliferation in both GBM cell lines

**Figure 4:**
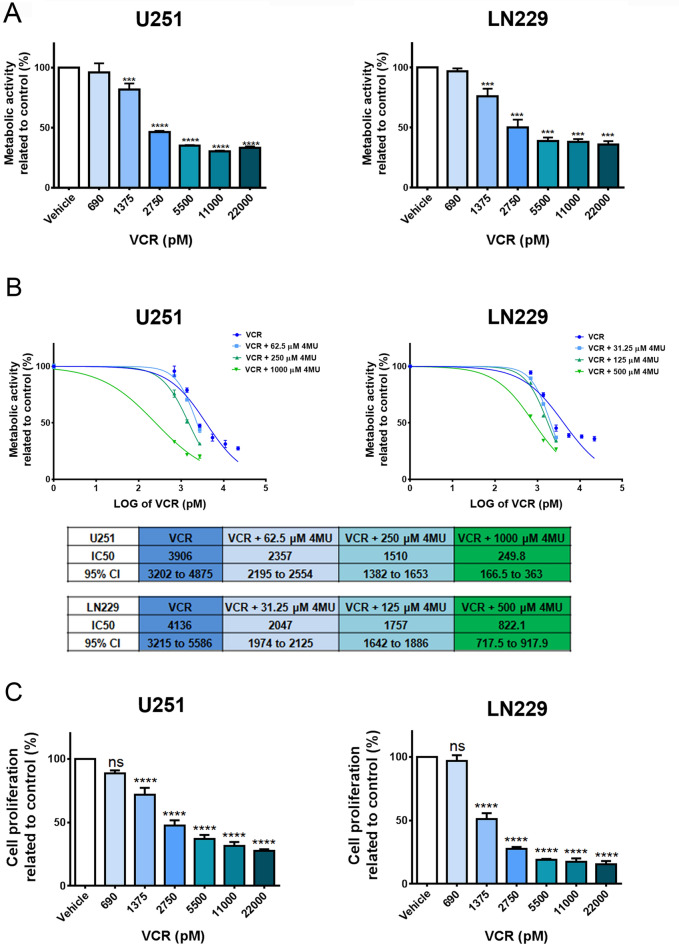

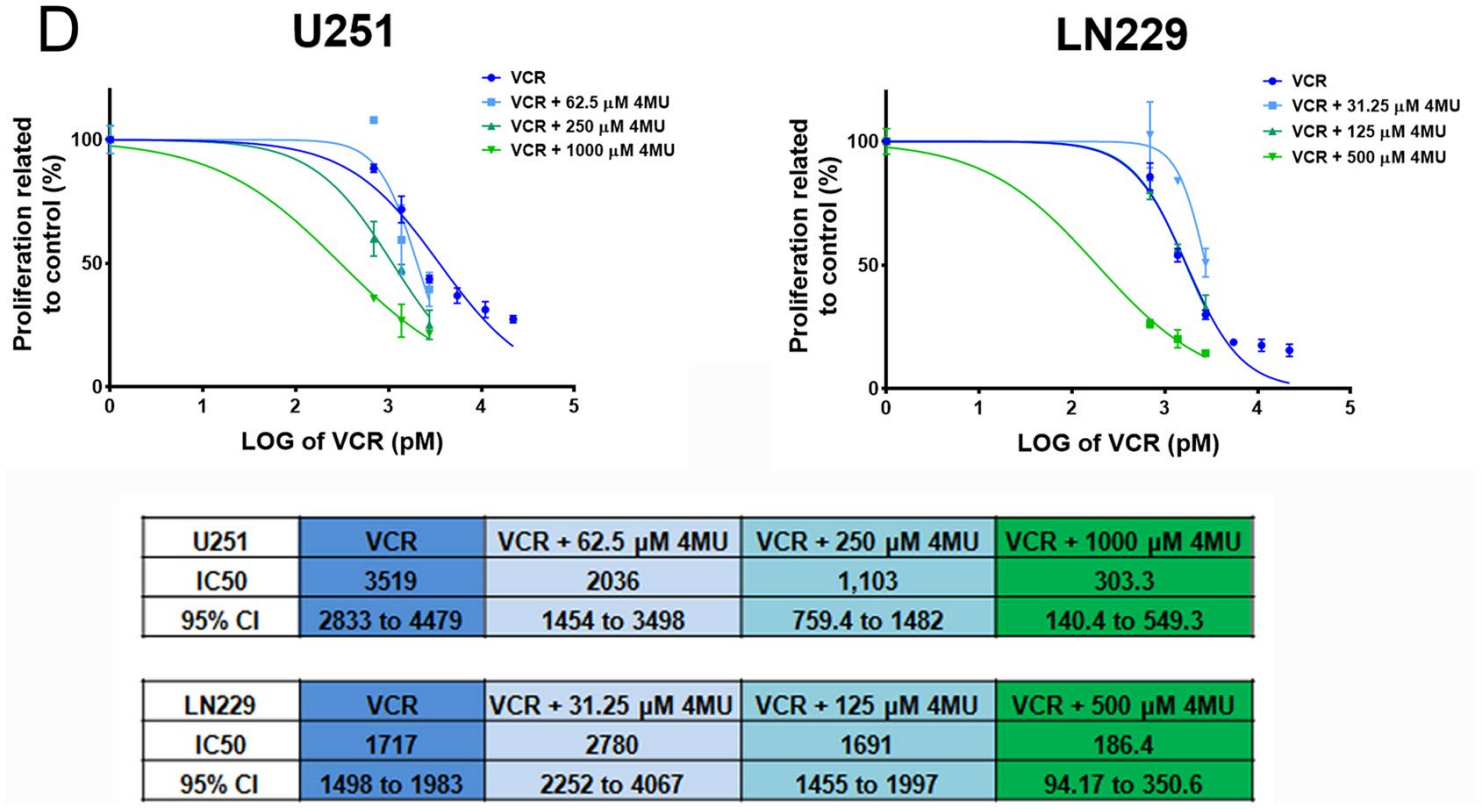
Effect of VCR and its combination with 4MU on metabolic activity and cell proliferation. **(A,B)** Metabolic activity was determined by XTT assay after 72 h of treatment with **(A)** VCR or **(B)** its combination with 4MU. Results are expressed as the percentage of Abs (n = 3) in relation to vehicle control, as described in the “Materials and methods” section. The dose–response curve is shown for the combined treatments, along with IC50 values for each treatment and its 95% CI. **(C,D)** Cell proliferation was determined by BrdU incorporation and ELISA-like assay after 72 h of treatment with **(C)** VCR or **(D)** its combination with 4MU. Results are expressed as the percentage of Abs (n = 3) in relation to vehicle control, as described in the “Materials and methods” section. The dose–response curve is shown for the combined treatments, along with IC50 values for each treatment and its 95% CI. In all graphs, each dot or bar represents the mean ± SD of at least 3 independent experiments. Asterisks (*) over each bar indicate differences between treated cells and vehicle control cells. *P < 0.05, **P < 0.01, ***P < 0.001, ****P < 0.0001 and ns = Non-significant (P > 0.05).

In the last years, VCR has been studied as a potential therapy in combination with other drugs for the treatment of GBM. Even though its benefits are still in debate, it is already being used as a therapeutic option in other brain tumors^11,13^. Considering this antecedent, and the fact that 4MU seems to be particularly useful in drug combination, we decided to investigate the effect of VCR individually or combined with 4MU in our study models. First, we assessed the effect of VCR on metabolic activity showing that, after 72 h of treatment, this drug decreased this parameter in a dose-dependent manner on both cell lines (Fig. 4A). Notably, the addition of 4MU sensitized both GBM cells to VCR, decreasing the IC50 by 10.5 and 3 times for U251 and LN229, respectively (Fig. 4B).

Then, we performed a BrdU incorporation assay and demonstrated that VCR markedly diminished cell proliferation in a dose-dependent manner (Fig. 4C). As expected, 4MU enhanced the effect of VCR on both cell lines, diminishing by 7.8 and 8.7 times the IC50 parameters on U251 and LN229 cell lines, respectively (Fig. 4D).

Overall, these results emphasize 4MU as a promising drug to be combined with others employed in the treatment of GBM and even in other pathologies that employ VCR as chemotherapy.

### 4MU exhibits antitumor effects on TMZ-resistant cells and sensitizes them to TMZ and VCR treatment

**Figure 5:**
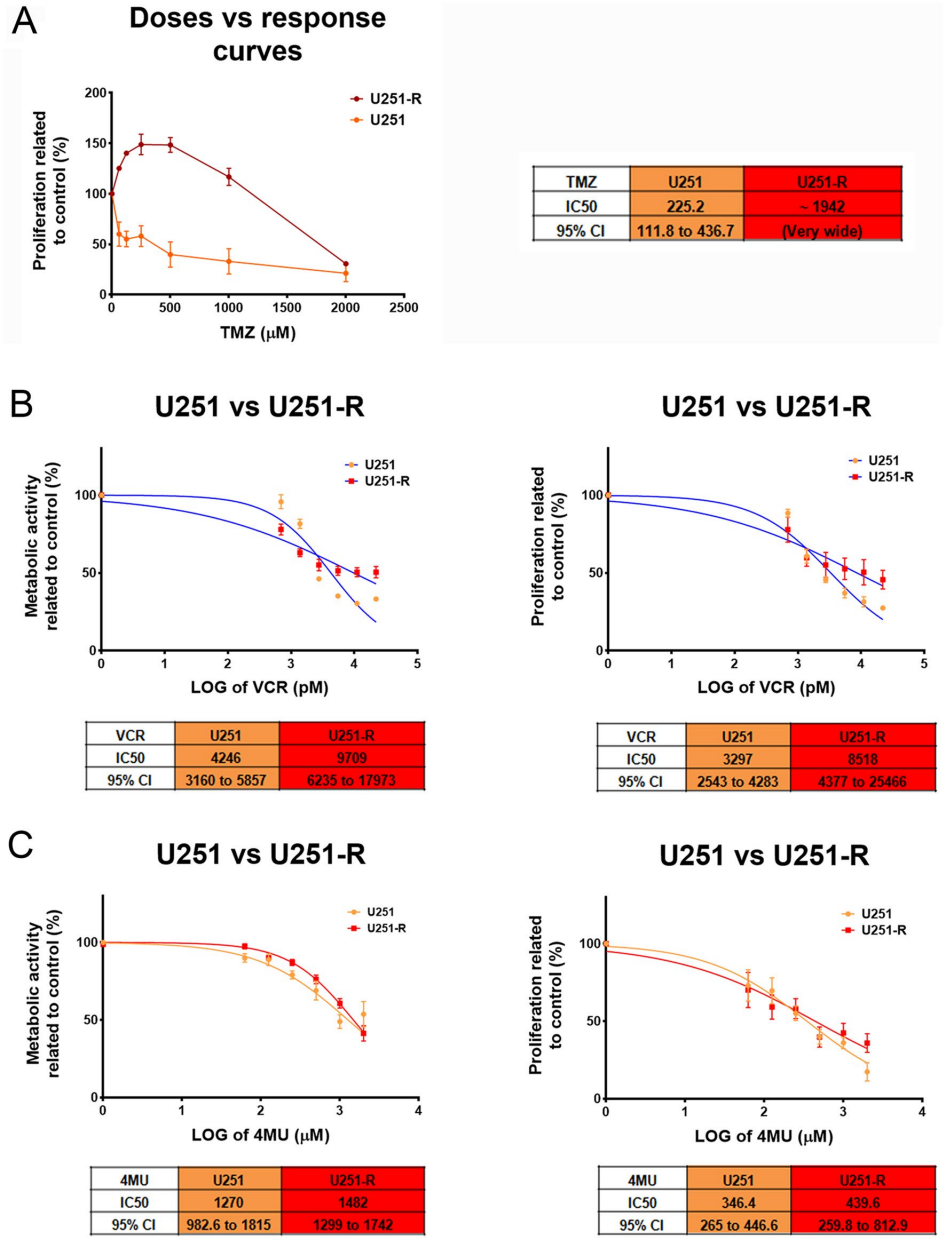

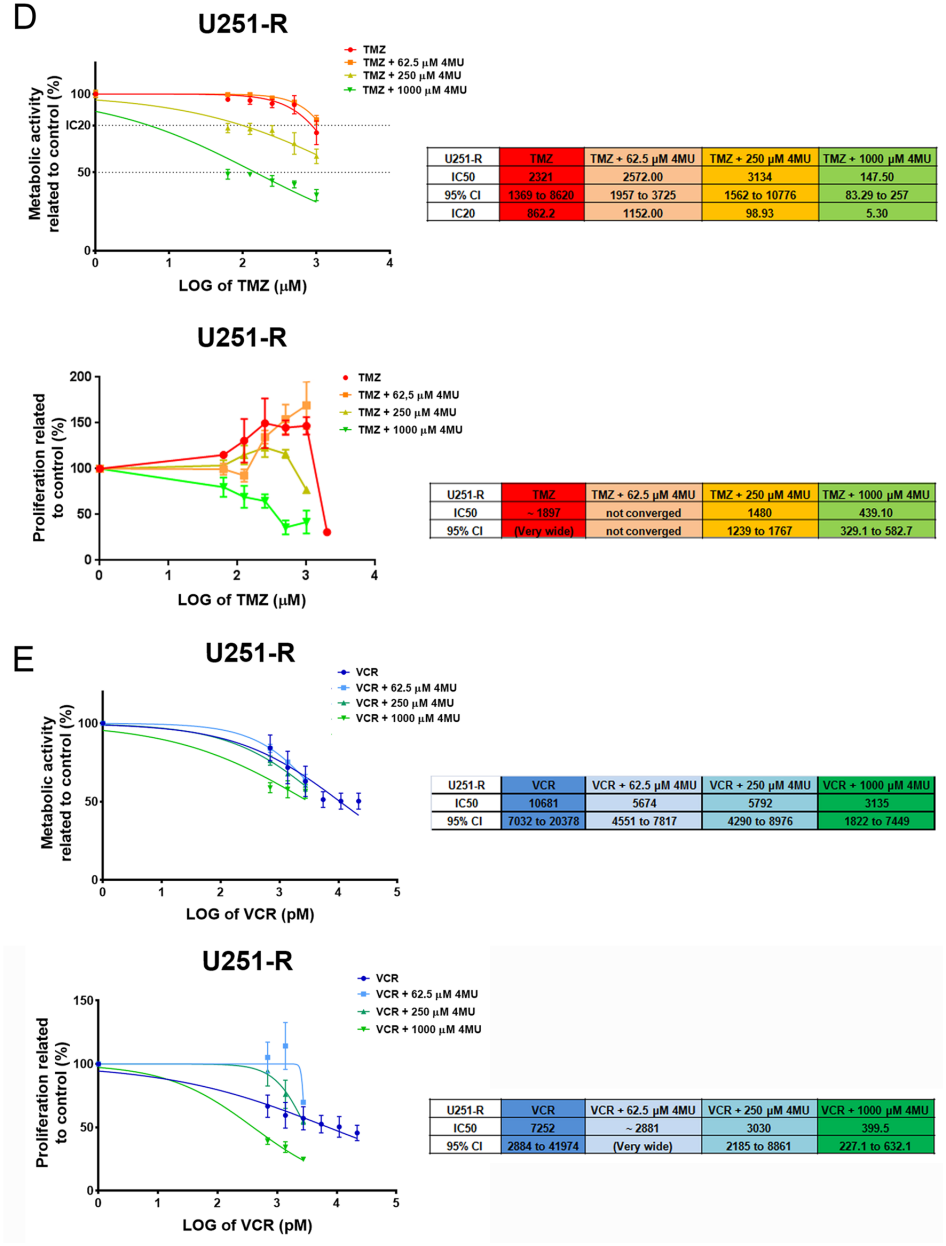
Effect of TMZ, VCR and their combination with 4MU on metabolic activity and cell proliferation of U251-R cells. **(A)** Cell proliferation was determined by BrdU incorporation and ELISA-like assay after 72 h of treatment with TMZ. Results are expressed as the percentage of Abs (n = 3) in relation to vehicle control, as described in the “Materials and methods” section. The dose–response curves are shown for U251 *wt* and TMZ-R cell lines, along with their IC50 values and its 95% CI. **(B–E)** Metabolic activity was determined by XTT assay and cell proliferation was determined by BrdU incorporation and ELISA-like assay after 72 h of treatment with **(B)** VCR, **(C)** 4MU, **(D)** TMZ plus 4MU or **(E)** VCR plus 4MU. Results are expressed as the percentage of Abs (n = 3) in relation to vehicle control, as described in the “Materials and methods” section. The dose–response curves are shown for U251 *wt* and TMZ-R cell lines, along with their IC50 values and its 95% CI. In all graphs, each dot represents the mean ± SD of at least 3 independent experiments.

As mentioned in the introduction, 50% of the patients with GBM present resistance to the first-line drug TMZ^10^. However, considering that recurrent GBM is also frequently presented as refractory to TMZ, this issue should be considered to a much greater extent. Therefore, the assessment of new therapeutic alternatives in a TMZ-resistant model becomes relevant. With this aim, we developed a U251-R cell line and evaluated its cell proliferation under TMZ treatment. After 72 h of treatment, U251-R cells present an IC50 of around 1950 μM to TMZ, while its parental *wild-type* (*wt*) counterpart has an IC50 of 225 μM (Fig. 5A), supporting the use of this cell line as a TMZ-resistant model. It is important to note that the resistant cell line, not only presents a higher IC50 of TMZ, but also enhances its proliferation in response to doses lower than 1000 μM, showing that TMZ is both ineffective as an antitumor treatment and somehow beneficial for the growth of such cells.

Notably, after 72 h of treatment with VCR, the dose–response curves obtained for metabolic activity and proliferation were statistically different (p < 0.05) from its parental cell line, which suggests that these cells also acquired cross-resistance to VCR (Fig. 5B). Remarkably, 4MU has the same effect on both U251 and U251-R cells on metabolic activity and cell proliferation, being the dose–response curves generated for both cell lines not statistically different (Fig. 5C). These results imply that the acquisition of resistance to TMZ had not impact on their sensitivity to 4MU effects. Regarding drug combination, 4MU sensitizes the U251-R cell line to the effect of TMZ on metabolic activity and cell proliferation (Fig. 5D), suggesting that 4MU would improve the response to TMZ even in TMZ-resistant cells. Similar results were obtained when combining 4MU with VCR (Fig. 5E). To reinforce these data we performed the same assays on LN229-R cell line, and obtained similar results (see Supplementary Fig S4). All in all, these results highlight the potential use of 4MU both alone and in combination with GBM-treatment drugs on patients who are refractory to first-line drugs.

## Discussion

In the last decades, research advances have improved cancer treatments. However, regarding brain tumors, and particularly GBM, there are few available therapies^14,43,44^. TMZ, an alkylating agent, is the first-line chemotherapy for this type of tumor, but it has several adverse effects, and notably, 50% of patients are refractory to this drug^6,8,9^. In this scenario, the search for new therapeutic alternatives has become a need to improve the prognostic and quality of life of GBM patients. Previously, we have demonstrated the effect of 4MU on murine and human GBM cell lines, and recently its effect in an orthotopic murine model has been reported^40–42^. In the present work, we show for the first time the beneficial effect of 4MU in combination with TMZ, as well as with VCR, on the human GBM cell lines U251 and LN229. Particularly, 4MU enhances the effect of TMZ on metabolic activity and proliferation on both cell lines, reducing the IC50 parameter several times compared to TMZ alone. Similar results were obtained in our previous work on the GL26 cell line^40^. Interestingly, we observed that 4MU also enhances the effect of TMZ on cell migration and MMP-2 activity, two processes closely related to GBM aggressiveness. Considering the lack of toxicity of 4MU^23,27^, and the in vitro selectivity index obtained in the present work, our results suggest not only an improvement in the antitumor effect of both drugs but could also enable a reduction of the dosage of TMZ, and consequently of its adverse effects, which would ultimately lead to a better quality of life for GBM patients. Notably, other authors that have used 4MU in combination with several chemotherapeutic drugs obtained similar results, which suggests that 4MU would be a promising drug as adjuvant therapy not only in GBM but also across a variety of tumors^34–46^. Furthermore, given that 4MU has been approved as a choleretic treatment in Europe and Asia, its use in GBM only represents a proposal for drug repositioning, which means that the time and research needed for it to reach patients should be significantly shorter than that for new drugs^47,48^. This is of relevance given the urgent need for more effective therapies for GBM patients.

Additionally, we assessed if 4MU was useful in combination with VCR, an emergent drug for GBM treatment^11,13^. Although VCR is already being used for other tumors, it also has important adverse effects, such as myelosuppression and peripheral neuropathy^49^. Therefore, as with TMZ, the combination with 4MU becomes relevant to reduce such effects. Firstly, we observed that VCR diminished metabolic activity and cell proliferation in a dose-dependent manner on both GBM cell lines. Similar results were obtained using several other GBM cells^50^. Notably, 4MU enhanced the effects of VCR on both mentioned processes, decreasing the dose of VCR needed to reach 50% of inhibition by about 8 times on both U251 and LN229 cells. In addition, we have previously shown the synergistic effect of this drug combination in a leukemia model^51^.

These results highlight the potential use of 4MU as co-adjuvant chemotherapy, not only with the first-line drug but also with emergent drugs, such as VCR, for GBM treatment.

Considering the high percentage of patients resistant to TMZ, we developed a model to study the effects of 4MU in this condition. For this purpose, we established a TMZ-resistant U251 cell line. This line showed an IC50 of around 1950 μM, 8.6-fold higher than the *wt* U251 cell line. It is worth noting that, apart from TMZ reduced efficacy in these cells, lower doses increased their proliferation. Similar results were obtained on the LN229-R cell line. Here, we report for the first time this effect of TMZ on the resistant GBM cell lines, which is of great interest if we consider that this effect might be the case for refractory patients. Therefore, treating such patients with the first-line drug would not only be ineffective but more importantly, could be detrimental to these patients. Further studies should be conducted to identify the key molecular players that are responsible for such response.

Regarding 4MU treatment, this drug still shows consistent inhibition of metabolic activity and cell proliferation on U251-R and LN229-R cells. Interestingly, these effects were similar to that obtained for their *wt* counterparts, meaning that the acquired mechanisms that confer resistance to both TMZ and VCR in resistant cells do not affect their sensitivity to 4MU. Moreover, 4MU prevented the TMZ-induced proliferation on resistant cells and even decreased their metabolic activity and cell proliferation, reaching similar IC50 values to that obtained with TMZ alone on *wt* cells, that is, almost fully reversing the TMZ-resistant phenotype. Interestingly, U251-R cells also acquired resistance to VCR, increasing the IC50 values by 2.5 times, which poses a multi-drug resistance phenotype, similar to that obtained by other authors on various types of tumors^45,52^. Moreover, 4MU counteracted the VCR resistance in the U251-R cells, restoring the IC50 values to those of the *wild-type* cell line. Similar results were obtained on LN229-R cell line and, previous to this work on leukemia models^45,51^. Therefore, the absence of resistance to 4MU on these cell lines suggests a completely different mechanism of action for the coumarin derivative, allowing it to be combined with both TMZ and VCR for better efficacy.

Many authors have attributed 4MU effects as a chemosensitizer to its ability to inhibit HA synthesis, hence preventing those tumor-promoting processes associated with higher HA content^22,34,30,46^. In this way, we previously demonstrated that HA activate Pgp and PI3K pathway as key chemoresistance mechanisms in CML cells, while 4MU abrogated HA levels and sensitized such cells to VCR and Imatinib effects^51,53^. Nevertheless, in these GBM models, our group has previously observed that 4MU exerts cytostatic effects independently of this inhibition^41^. Moreover, Díaz et al. showed in an acute leukemia model that 4MU inhibitory effects still occurred even without the inhibition of cell HA synthesis, as well as in cells that do not synthesize HA^28^. Notably, 4MU sensitization to radiotherapy and vemurafenib was reported in a fibrosarcoma and melanoma model, respectively^36,54^. These reports are part of an increasing amount of evidence regarding 4MU as an oxidative stressor and metabolic reprogramming agent, rather than just an inhibitor of HA synthesis^54–56^. Particularly in GBM, TMZ treatment enhances ROS production, which contributes to the induction of senescence and apoptosis^57^. However, chemoresistance to the first-line drug, even in MGMT-independent resistance, has been associated with enhanced antioxidant pathways and co-treatment of those models with pro-oxidants proved to be effective in reversing TMZ resistance^58,59^. Taking these reports into account, we propose that 4MU may act at the metabolic level in GBM cell lines. This could explain 4MU induction of senescence^41^ and its chemo-sensitizing properties, even in TMZ-resistant cells, when combined with TMZ or VCR, which have very different mechanisms of action. Future studies should attempt to identify the metabolic mechanisms underlying these effects to provide a better insight into the potential use of 4MU as adjuvant chemotherapy.

## Conclusion

In conclusion, our results support 4MU as a potential drug in the therapy of GBM, both alone and in combination with the first-line drug, even in TMZ-resistant GBM.

## Materials and methods

### Reagents

4-Methylumbelliferone (4MU), propidium iodide (PI), fluorescein diacetate (FDA), 4ʹ,6-diamidino-2-phenylindole (DAPI), gelatin, glucose, BSA, XTT and phenazine methosulfate (PMS) were purchased from Sigma-Aldrich (Saint Luis, Missouri, USA). DMEM, l-glutamine, streptomycin and penicillin were purchased from Invitrogen (Waltham, Massachusetts, USA). SFB was purchased from Natocor (Argentina). BrdU, monoclonal mouse anti-BrdU (317902) antibody, and goat anti-mouse HRP (405306) secondary antibody were purchased from Biolegend (San Diego, CA, USA). The goat anti-mouse cy3 (115-165-003) secondary antibody was obtained from Jackson Immunoresearch (West Grove, Pennsylvania, USA) and Mowiol (Calbiochem) was purchased from Merck S.A (Buenos Aires, Argentina).

### Cell cultures

The LN229 and U251 human GBM cell lines (gently provided by Dr. C. Perez-Castro and Dr. M. Candolffi, respectively) were cultured at 37 °C in a 5% CO_2_ atmosphere with DMEM supplemented with 10% heat inactivated fetal bovine serum (FBS), 2 mM l-glutamine, 100 µg/ml streptomycin and 100 IU/ml penicillin (DMEM-C) and tested for Mycoplasma every three months by DAPI staining. Cells passages lower than 20 were used for the described experiments.

### Normal brain primary cultures

The murine normal brain primary cultures (NBPC) were generated from C57BL/6 mice as previously described^40^. Animal procedures followed the guidelines from our Laboratory Animal Welfare Committee CICUAL-Facultad de Farmacia y Bioquímica-Universidad de Buenos Aires (RES (D) No 4538/2018). Briefly, 2–4 day-old mice were euthanized by decapitation and brains were resected. Cerebral hemispheres were mechanically dissociated in DMEM/F12 medium. The largest tissue fragments were allowed to spontaneously settle, while the upper homogenous cell suspension was recovered and centrifuged. The pellet was resuspended and cells were cultured in DMEM/F12 supplemented with 10% and 20 µg/ml streptomycin and 20 IU/ml penicillin FBS at 37 °C in a 5% CO2 atmosphere.

### U251-R and LN229-R cells establishment and culture

To generate the stable U251-R and LN229-R cell lines, the wild-type U251 and LN229 cell lines were grown in DMEM-C with sequential increments of TMZ as was previously reported^60^. Once reached 200 µM of TMZ, the IC50 was assessed through XTT and BrdU incorporation assays to corroborate the increment in such parameter. Once resistance was established, the cells were continuously maintained in DMEM with 200 µM of TMZ.

### Cell treatments

For all assays, cells were seeded 24 h before treatment. Cells were treated with either TMZ (0–2000 µM), VCR (0–20 nM), or 4MU (0–1000 µM), or a combination of them as appropriate. Untreated or vehicle control cultures were also included. All incubations were performed at 37 °C in a 5% CO_2_ atmosphere.

### XTT assay

For the XTT assay, 3 × 10^3^ cells/well were seeded in 96-well plates and treated with 4MU, and/or TMZ or VCR for 48 h. After treatment, culture medium was discarded and 100 µl of an XTT solution (1 mg/ml) containing PMS (7.5 µg/ml) was added to each well. Cells were incubated for two additional hours at 37 °C in a 5% CO_2_ atmosphere. After incubation, the absorbance (Abs) was read at 450 nm and 620 nm using a microplate reader (Multiscan Ex, Absorbance Microplate Reader, Thermo Electron Corporation, China). Cell viability was calculated as:

$$\left[ {{\text{Ab }}\left( {{\text{treated}}} \right)_{{{45}0}} {-}{\text{ Ab }}\left( {{\text{treated}}} \right)_{{{62}0}} /{\text{ Ab }}\left( {{\text{untreated}}} \right)_{{{45}0}} {-}{\text{Ab }}\left( {{\text{untreated}}} \right)_{{{62}0}} } \right] \times { 1}00.$$

The in vitro Selectivity Index (SI) was calculated as:

$$\left[ {{\text{IC5}}0{\text{ of TMZ}}/{\text{4MU treatment}}} \right]_{{\text{NBPC cells}}} , / \, \left[ {{\text{ IC5}}0{\text{ of TMZ}}/{\text{4MU treatment}}} \right]_{{\text{U251 cells}}}$$

and

$$\left[ {{\text{IC3}}0{\text{ of TMZ}}/{\text{4MU treatment}}} \right]_{{\text{NBPC cells}}}, / \, \left[ {{\text{ IC3}}0{\text{ of TMZ}}/{\text{4MU treatment}}} \right]_{{\text{LN229 cells}}}$$

### Cell proliferation

Briefly, 3 × 10^3^ cells/well were seeded in 96-well plates and treated for 46.5 h with 4MU, TMZ, VCR or their combinations. Then, BrdU was added at final a concentration of 20 µM and cells were incubated for 2 h. After this time, the supernatant was removed, the cells were washed and fixed with PFA 4% for 20 min and permeabilized with HCl 2N. Then, cells were neutralized with sodium tetraborate (0.1 M; pH 9) and the endogenous peroxidase activity was blocked with 3% H_2_O_2_ in methanol for 30 min at room temperature. After that, cells were blocked with SFB 2% O.N. and incubated with mouse anti-BrdU antibody (1/1000) O.N. at 4 °C. Finally, HRP conjugated anti-mouse antibody was added (1/2000) and incubated for 2 h at room temperature. The plate was revealed with TMB and the reaction was stopped after 10 min with 4N H_2_SO_4_. Absorbance was read at 450 nm and 620 nm using a microplate reader (Multiscan Ex, Absorbance Microplate Reader, Thermo Electron Corporation, China). Cell proliferation was calculated as:

$$\left[ {{\text{Ab }}\left( {{\text{treated}}} \right)_{{{45}0}} {-}{\text{ Ab }}\left( {{\text{treated}}} \right)_{{{62}0}} /{\text{ Ab }}\left( {{\text{untreated}}} \right)_{{{45}0}} {-}{\text{Ab }}\left( {{\text{untreated}}} \right)_{{{62}0}} } \right] \times { 1}00.$$

### Cell death

Propidium iodide (PI) staining was performed as previously described^40,61^. Briefly, 3 × 10^5^ cells/well were seeded in 12-well plates and treated for 72 h. Cells were then stained with fluorescein diacetate FDA (1.4 µM) for 20 min, harvested, centrifuged and washed. Subsequently, the cell pellet was resuspended and incubated with PI (5 μg/ml) for 5 min. Stained cells were acquired on a Pas III flow cytometer (Partec, Germany) and analyzed with the Flowing 2.1.5 software (Scripps Institute, La Jolla, USA).

### Zymography

The MMPs activity was evaluated by gelatin zymography as previously described^40,41^. Briefly, 2 × 10^5^ cells/well were incubated in 48-well plates with serum-free DMEM supplemented with 0.01% BSA and 0.1% glucose with or without each treatment. After 24 h, supernatants were centrifuged and loaded on 7.5% SDS-PAGE gels containing gelatin (1 mg/ml). A molecular weight marker was run in parallel. After electrophoresis, gels were washed three times with 2.5% Triton X-100 for 20 min, and then incubated with 25 mM Tris–HCl pH 7.5; 5 mM CaCl_2_; 0.9% NaCl; 0.05% NaN_3_ for 48 h at 37 °C. The gelatinolytic activity was revealed by staining with 0.5% Coomassie blue. Photographs were obtained with a BioSpectrum® 515 Imaging System M-26XV (UVP, Cambridge, UK) and analyzed with the Image J software.

### Wound healing assay

Migration assay was performed as previously described, with slight modifications^41^. Cells were seeded in 24-well plates until they reached confluence. The monolayer was then scratched with a 200 µl sterile pipette tip and incubated in DMEM containing 3% SFB with or without treatment. The same wound area was photographed at 24 h. The Image J software was used to calculate wound area.

Results were expressed as migration index calculated as

$${\text{Closure gap index }} = \, \left[ {\left( {{\text{area}}} \right)_{{{\text{t}} = 0{\text{h}}}} - \, \left( {{\text{area}}} \right)_{{{\text{t}} = {\text{24h}}}} } \right]_{{{\text{treated}}}} / \, \left[ {\left( {{\text{area}}} \right)_{{{\text{t}} = 0{\text{hs}}}} - \, \left( {{\text{area}}} \right)_{{{\text{t}} = {\text{24h}}}} } \right]_{{{\text{control}}}} .$$

### Statistical analysis

Statistical significance was calculated using Prism 7 statistical software (Graph Pad Prism, San Diego, CA, USA). The data presented in this study is expressed as mean values ± SD if not otherwise stated. The letter “n” refers to the number of independently performed experiments corresponding to the data shown in each figure. This number was selected in each case considering the statistic test and the capability of the laboratory and it is shown in the captions of each figure. Shapiro–Wilk´s normality test was performed prior to the statistical test. The variances were analyzed within each group of data by Levene’s test and were similar between the groups that were statistically compared. One way-ANOVA was used to compare three or more independent groups. The Student’s test (T-test) was performed to compare two independent groups whereas for comparison of treated vs. untreated groups Dunnet’s test was performed. Bonferroni’s test was used to make simultaneous multiple comparisons between different groups. P values < 0.05 were considered statistically significant.

## Supplementary Information

Supplementary Figures 1–3.

Supplementary Figure 4.

## Data Availability

The datasets generated during and/or analysed during the current study are available from the corresponding author on reasonable request.
