# Supplementary material for: 4-Methylumbelliferone enhances the effects of chemotherapy on both temozolomide-sensitive and resistant glioblastoma cells
Source: Sci Rep. 2023 Jun 8;13:9356. doi: 10.1038/s41598-023-35045-3 (PMC10249561; doi:10.1038/s41598-023-35045-3)
Supplement: Supplementary file 1 — Supplementary Figures 1–3. [file 41598_2023_35045_MOESM1_ESM.docx]

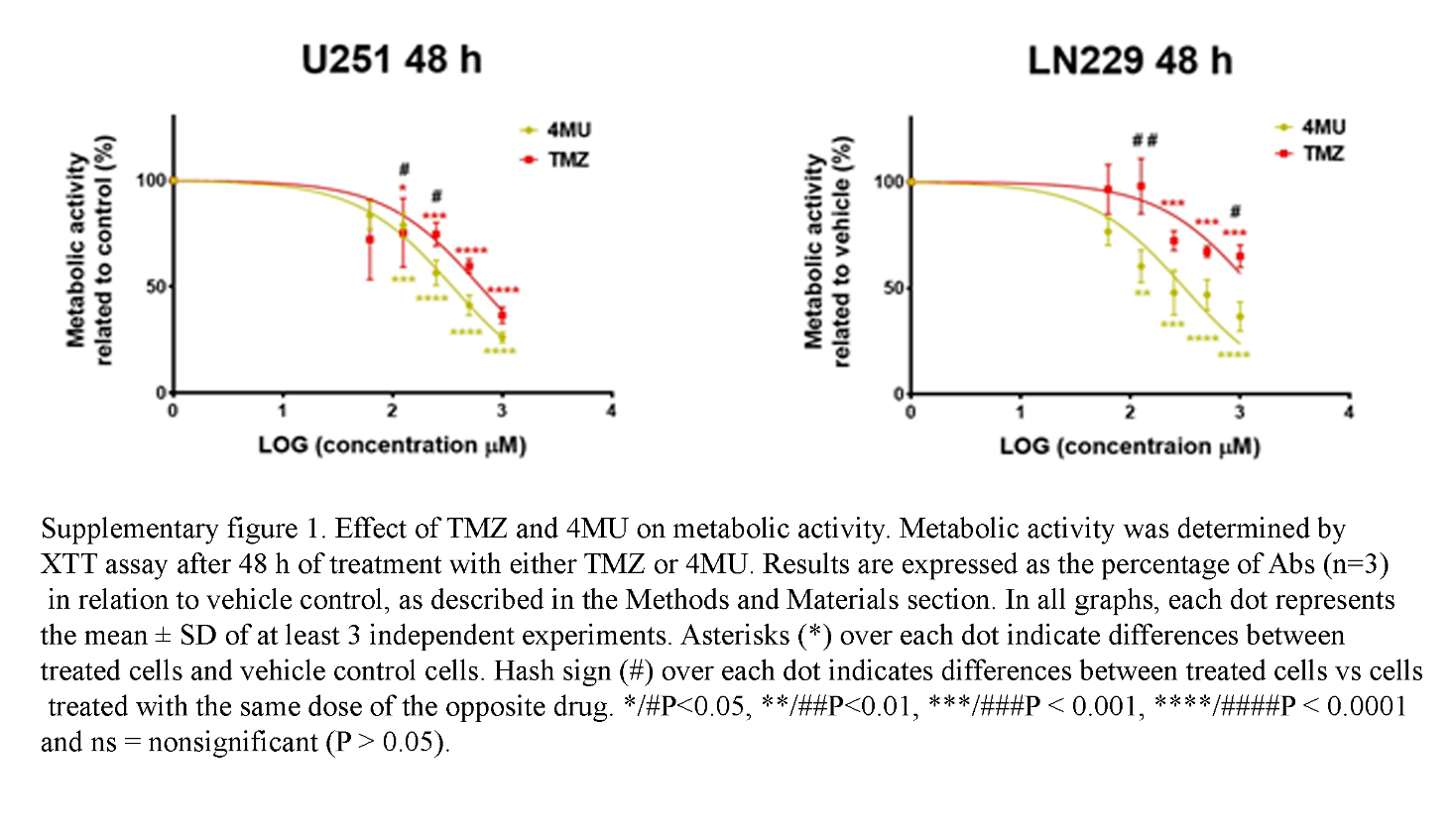


*Supplementary figure 1. Effect of TMZ and 4MU on metabolic activity.* Metabolic activity was determined by XTT assay after 48 h of treatment with either TMZ or 4MU. Results are expressed as the percentage of Abs (n=3) in relation to vehicle control, as described in the Methods and Materials section. In all graphs, each dot represents the mean ± SD of at least 3 independent experiments. Asterisks (*) over each dot indicate differences between treated cells and vehicle control cells. Hash sign (#) over each dot indicates differences between treated cells vs cells treated with the same dose of the opposite drug. */#P<0.05, **/##P<0.01, ***/###P < 0.001, ****/####P < 0.0001 and ns = nonsignificant (P > 0.05).


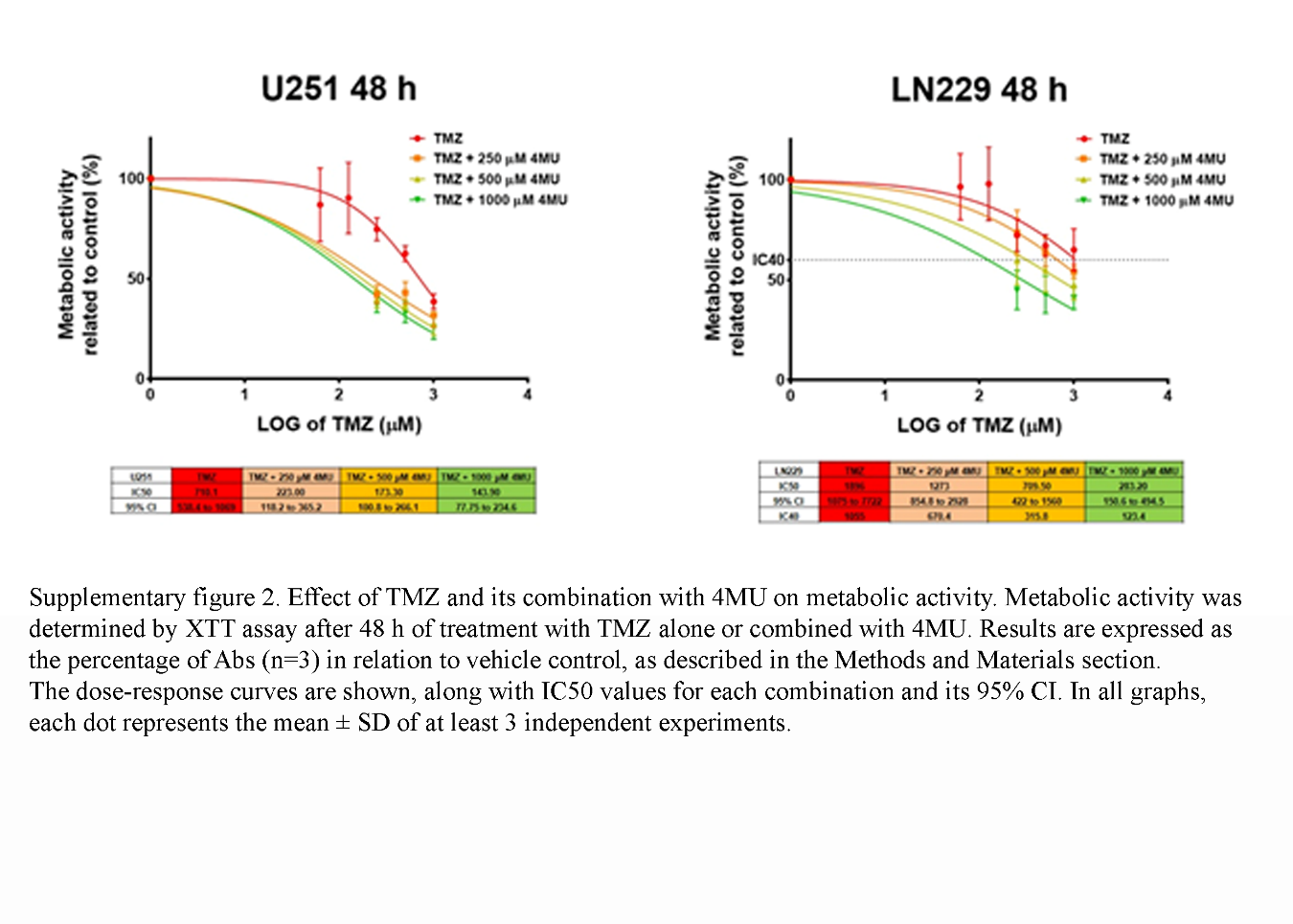


*Supplementary figure 2.* *Effect of TMZ and its combination with 4MU on metabolic activity.* Metabolic activity was determined by XTT assay after 48 h of treatment with TMZ alone or combined with 4MU. Results are expressed as the percentage of Abs (n=3) in relation to vehicle control, as described in the Methods and Materials section. The dose-response curves are shown, along with IC50 values for each combination and its 95% CI. In all graphs, each dot represents the mean ± SD of at least 3 independent experiments.

*Supplementary figure 3. Effect of TMZ and its combination with 4MU on proliferation. The* proliferation was determined by BrdU incorporation after 24 h of treatment with TMZ alone or combined with 4MU in DMEM with 3% FBS. Results are expressed as the percentage of Abs (n=3) in relation to vehicle control, as described in the Methods and Materials section. The dose-response curves are shown. In all graphs, each dot represents the mean ± SEM of at least 3 independent experiments.
