# Supplementary material for: 4-Methylumbelliferone enhances the effects of chemotherapy on both temozolomide-sensitive and resistant glioblastoma cells
Source: Sci Rep. 2023 Jun 8;13:9356. doi: 10.1038/s41598-023-35045-3 (PMC10249561; doi:10.1038/s41598-023-35045-3)
Supplement: Supplementary file 2 — Supplementary Figure 4. [file 41598_2023_35045_MOESM2_ESM.docx]

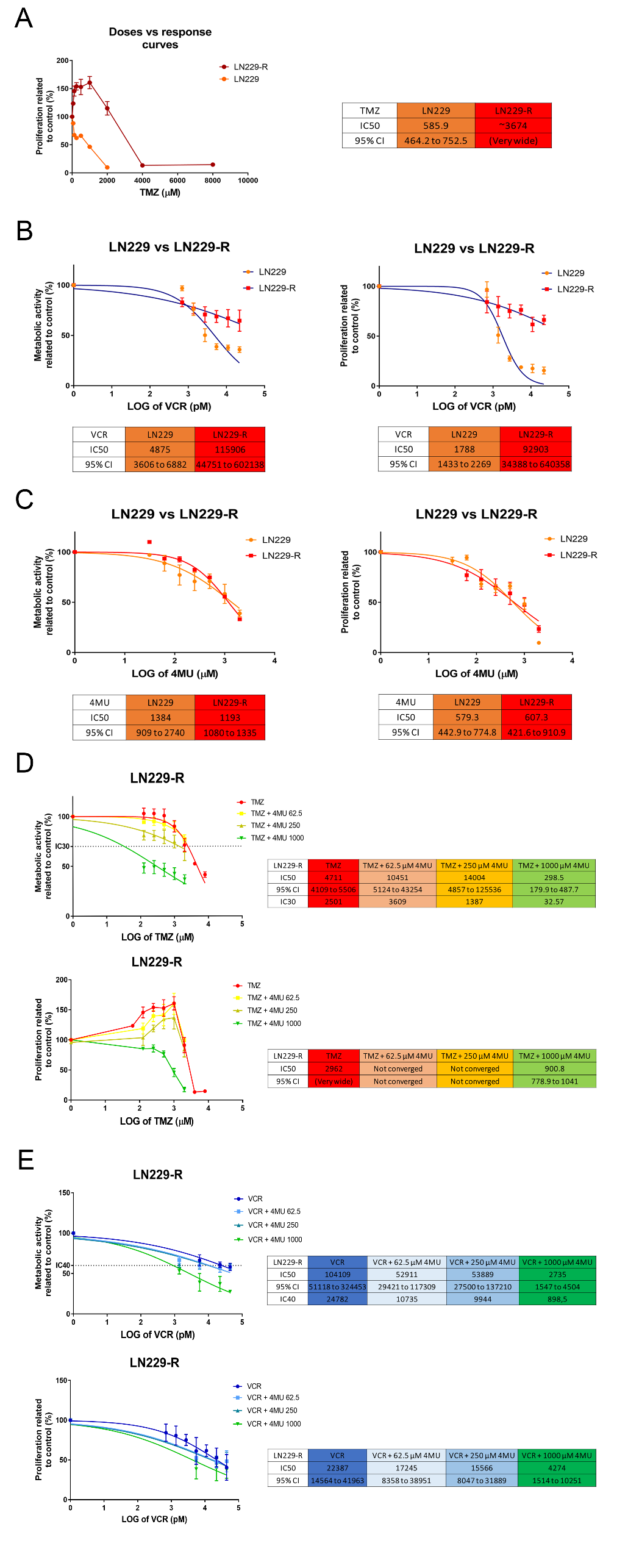


**Figure Sup. 4. Effect of TMZ, VCR and their combination with 4MU on metabolic activity and cell proliferation of LN229-R cells. (A)** Cell proliferation was determined by BrdU incorporation and ELISA-like assay after 72 h of treatment with TMZ. Results are expressed as the percentage of Abs (n=3) in relation to vehicle control, as described in the Method and Materials section. The dose-response curves are shown for LN229 *wt* and TMZ-R cell lines, along with their IC50 values and its 95% CI. **(B-E)** Metabolic activity was determined by XTT assay and cell proliferation was determined by BrdU incorporation and ELISA-like assay after 72 h of treatment with **(B)** VCR, **(C)** 4MU, **(D)** TMZ plus 4MU or **(E)** VCR plus 4MU. Results are expressed as the percentage of Abs (n=3) in relation to vehicle control, as described in the Methods and Materials section. The dose-response curves are shown for LN229 *wt* and TMZ-R cell lines, along with their IC50 values and its 95% CI. In all graphs, each dot represents the mean ± SD of at least 3 independent experiments.
